# Factors associated with cognitive impairment before intracerebral haemorrhage: community-based neuropathological study

**DOI:** 10.1093/braincomms/fcae275

**Published:** 2024-08-22

**Authors:** Yawen Xiang, Mark A Rodrigues, Christine Lerpiniere, Tom J Moullaali, James J M Loan, Tim Wilkinson, Catherine A Humphreys, Colin Smith, Rustam Al-Shahi Salman, Neshika Samarasekera

**Affiliations:** Centre for Clinical Brain Sciences, University of Edinburgh, Edinburgh EH16 4SB, UK; Centre for Clinical Brain Sciences, University of Edinburgh, Edinburgh EH16 4SB, UK; Department of Neuroradiology, NHS Lothian, Edinburgh EH16 4SA, UK; Centre for Clinical Brain Sciences, University of Edinburgh, Edinburgh EH16 4SB, UK; Centre for Clinical Brain Sciences, University of Edinburgh, Edinburgh EH16 4SB, UK; Faculty of Medicine, The George Institute for Global Health, University of New South Wales, Sydney, NSW 2042, Australia; Centre for Clinical Brain Sciences, University of Edinburgh, Edinburgh EH16 4SB, UK; Centre for Clinical Brain Sciences, University of Edinburgh, Edinburgh EH16 4SB, UK; Usher Institute, University of Edinburgh, Edinburgh EH8 9AG, UK; Centre for Clinical Brain Sciences, University of Edinburgh, Edinburgh EH16 4SB, UK; Centre for Clinical Brain Sciences, University of Edinburgh, Edinburgh EH16 4SB, UK; Centre for Clinical Brain Sciences, University of Edinburgh, Edinburgh EH16 4SB, UK; Centre for Clinical Brain Sciences, University of Edinburgh, Edinburgh EH16 4SB, UK

**Keywords:** neuropathology, Alzheimer’s disease, intracerebral haemorrhage, small vessel disease

## Abstract

Little is known about whether clinical, radiological or neuropathological features are associated with cognitive impairment before intracerebral haemorrhage. We conducted a community-based cohort study of 125 adults with intracerebral haemorrhage (lobar *n* = 71, non-lobar *n* = 54) with consent to brain autopsy. We compared small vessel disease biomarkers on diagnostic CT head and neuropathological findings including neurofibrillary tangles and amyloid plaques in adults without cognitive impairment versus cognitive impairment without dementia versus dementia before intracerebral haemorrhage, stratified by lobar and non-lobar intracerebral haemorrhage. In non-lobar intracerebral haemorrhage, severe cortical atrophy was less common in those without cognitive impairment (8/36, 22%) and cognitive impairment without dementia (0/9, 0%) versus dementia (5/9, 56%); *P* = 0.008. Irrespective of intracerebral haemorrhage location, adults without cognitive impairment had milder neurofibrillary tangle pathology measured by median Braak stage (lobar intracerebral haemorrhage: no cognitive impairment 2 [interquartile range, 2–3] versus cognitive impairment without dementia 4 [2–6] versus dementia 5.5 [4–6]; *P* = 0.004; non-lobar intracerebral haemorrhage: no cognitive impairment 2 [1–2] versus cognitive impairment without dementia 2 [1–2] versus dementia 5 [3–6]; *P* < 0.001). Irrespective of intracerebral haemorrhage location, adults without cognitive impairment had milder amyloid plaque pathology measured by median Thal stage (lobar intracerebral haemorrhage: no cognitive impairment 2 [1–2] versus cognitive impairment without dementia 2 [2–3] versus dementia 2.5 [2–3.5]; *P* = 0.033; non-lobar intracerebral haemorrhage: no cognitive impairment 1 [0–1] versus cognitive impairment without dementia 0 [0–2] versus dementia 3 [2–3]; *P* = 0.002). Our findings suggest that irrespective of intracerebral haemorrhage location, adults with cognitive impairment before an intracerebral haemorrhage have more Alzheimer’s disease neuropathologic change.

## Teaser

Xiang *et al*. undertook a community-based cohort study to ascertain whether certain clinical, radiological or neuropathological features are associated with cognitive impairment before intracerebral haemorrhage. The study found that irrespective of intracerebral haemorrhage location, adults with cognitive impairment before an intracerebral haemorrhage have more Alzheimer’s disease neuropathologic change.

## Graphical Abstract

**Graphical Abstract:**
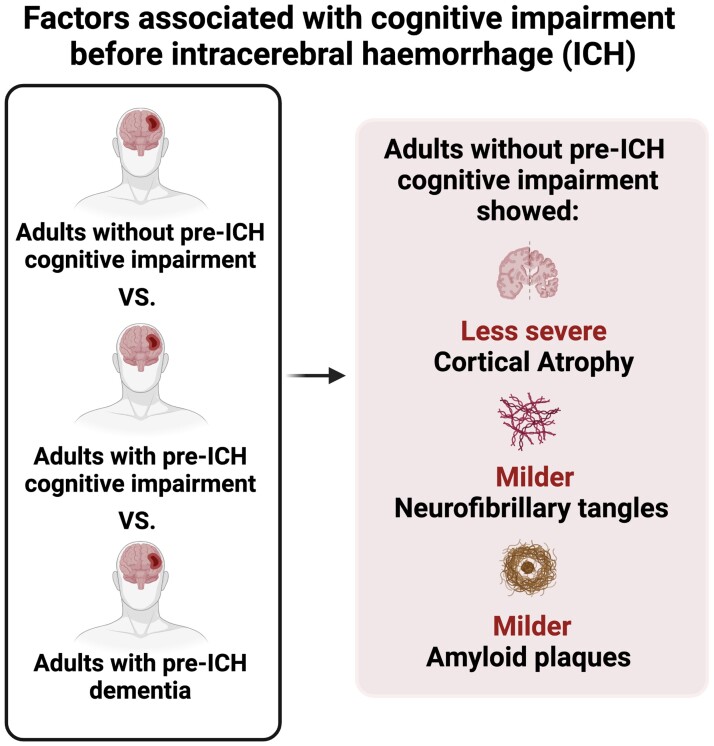

## Introduction

Spontaneous (non-traumatic) intracerebral haemorrhage (ICH) accounts for 9–27% of all strokes globally.^1^ ICH and dementia are closely linked, with about one-third of adults having cognitive impairment or dementia before their first ICH.^2^ About 85% of ICHs are attributed to small vessel disease (SVD), most commonly non-cerebral amyloid angiopathy (CAA) SVD (including arteriolosclerosis) and CAA.^3^ These are typically classified as lobar (affecting the cortical–subcortical areas of the cerebral hemispheres) or non-lobar (supratentorial deep ICH or infratentorial ICH). Hypertension is the most common attributable risk factor for both lobar and non-lobar ICH^4^ and may be a driver of arteriolosclerosis,^5^ which may explain the link between SVD and ICH.^6^ CAA, characterized by deposition of amyloid-beta protein within leptomeningeal and cortical blood vessels is associated with lobar ICH^7-9^ and Alzheimer’s disease.^10^ Either or both of the vasculopathies responsible for ICHs commonly occur in people with dementia,^3^ and the underlying SVD pathology could drive both the cognitive impairment and the ICH.

Increasing our understanding of the clinical, radiological and neuropathological features underlying cognitive impairment or dementia before an ICH could help us to target interventions for patients who are at risk of both ICH and dementia. It could also help us to understand how cognitive impairment before ICH contributes to cognitive impairment after ICH and dementia.^11^ It is commonly thought that the vasculopathies underlying dementia before an ICH are probably linked to the underlying SVD(s) which caused the ICH. Only seven hospital-based case series studies^12-18^ of 67 adults (lobar ICH *n* = 48, non-lobar ICH *n* = 1, multiple ICH *n* = 1, corpus callosum *n* = 1, unknown *n* = 16) have used post-mortem or biopsy specimens to examine brain vasculopathies in adults with dementia before an ICH compared to adults without dementia before their ICH; 65 (97%) adults had CAA but these studies did not systematically assess non-CAA SVD or compare imaging SVD biomarkers in those without cognitive impairment versus those with cognitive impairment or dementia before ICH.

We hypothesized that Alzheimer’s disease neuropathologic change [neurofibrillary tangles (NFT) and amyloid plaques (AP)] would be associated with lobar ICH and non-CAA SVD associated with non-lobar ICH.^18-21^

We aimed to determine whether clinical, radiological or neuropathological features differ between those without cognitive impairment versus cognitive impairment without dementia versus dementia before ICH, stratified by ICH location (lobar versus non-lobar ICH).

## Materials and methods

### Study design and participants

We did a prospective community-based inception cohort study of consecutive adults (aged 16 and above) with a first-ever spontaneous (non-traumatic) ICH in the Lothian health board region of Scotland (LINCHPIN study; mid-2010 population of Lothian health board region aged ≥ 16 years was 695 335). We prospectively identified adults with first-ever spontaneous ICH using multiple overlapping sources of case ascertainment.^22^ We excluded adults with an ICH secondary to trauma, macrovascular causes, structural causes or haemorrhagic transformation of an ischaemic stroke. We sought to enrol consecutive adults for research autopsy limited to the brain in case of death to examine the nature of SVD in adults with ICH. We have described the reasons why adults declined brain tissue donation elsewhere.^23^ The three most frequent reasons for declining participation were: (i) that the nearest relative was unable to decide on whether they should give consent on their relative’s behalf, (ii) the participant did not wish to be involved in a research study, and (iii) brain tissue donation was ‘too invasive’. There were no differences in age, sex or measures of ICH severity between those who consented to brain tissue donation and those who did not.^23^

If the patient lacked mental capacity as defined by the Adults with Incapacity (Scotland) Act 2000, we sought consent for brain tissue donation from their nearest relative or legal representative in accordance with the statutory requirements of the Human Tissue (Scotland) Act 2006 which requires consent to be sought before the use of a deceased person’s organs, tissues or cells for medical research. The median time interval between the date of ICH onset and the autopsy discussion was 5 days and 49% of those approached consented to autopsy.^23^ This study was approved by the Scotland A Research Ethics Committee (10/MRE00/23) and carried out in accordance with the Declaration of Helsinki. We obtained written consent from all participants or their next-of-kin when they lacked mental capacity.

### Clinical and radiological variables

We collected demographic and clinical information (including past medical history, medications and pre-morbid cognitive function) by interviewing participants or their next-of-kin, or by reviewing medical records at the time of presentation. We classified cognitive function blinded to radiological and neuropathological findings and evaluated adults as having dementia before their ICH if one or more of the following criteria were met at the time of presentation with ICH: (i) evidence that DSM-V criteria^24^ for major neurocognitive disorder (dementia) fulfilled by reviewing hospital or primary care records, (ii) cholinesterase inhibitor prescribed, and (iii) participants with a short form of the Informant Questionnaire on Cognitive Decline in the Elderly (IQCODE) > 64^25^ which had been completed by a close relative. When considering the management of dementia including starting cholinesterase inhibitors, prescribers in Scotland can follow the National Institute for Health and Clinical Excellence recommendations^26^ which recommend prescribing cholinesterase inhibitors in mild to moderate Alzheimer’s disease dementia or Scottish Intercollegiate Guideline Network Guidelines^27^ which recommend cholinesterase inhibitors for dementia of any severity. We classified adults as having cognitive impairment without dementia if there was evidence that DSM-V criteria for mild neurocognitive disorder (mild cognitive impairment) were fulfilled by reviewing hospital or primary care records. Two neurologists (T.W. and N.S) independently reviewed the medical records of all adults identified as having dementia before their ICH to determine the likely dementia syndrome. We reviewed records blinded to ICH location, CT SVD scores and neuropathological findings. Potential syndrome diagnoses included: Alzheimer’s disease, vascular dementia or mixed dementia (meeting Alzheimer’s disease and vascular dementia criteria), dementia with Lewy bodies, Parkinson’s disease dementia or frontotemporal dementia. We used a standard proforma with diagnostic criteria provided (Supplementary Appendix 1 and Appendix 2) to diagnose dementia based on a person’s clinical history, cognitive assessment and brain imaging done prior to their ICH.^28^ Given that in clinical practice people may be diagnosed with dementia without fulfilling formal criteria there was the option, both for the diagnosis of dementia and for the diagnosis of the dementia syndrome, to select a ‘formal criteria not met but diagnosis likely’ option.^28^

One neuroradiologist (M.A.R.) reformatted the diagnostic (first) non-contrast brain CT into standard axial, coronal and sagittal planes and used a standardized proforma derived from large-scale stroke studies to assess the images as previously described.^29^ M.A.R evaluated ICH location,^30^ the ICH volume using the ABC/2 method^31^ and CT biomarkers of cerebral SVD including anterior and posterior white matter lucencies (WML), lacunes, central and cortical atrophy using a standardized rating proforma.^32^ M.A.R calculated the CT SVD sum score^33^ by awarding one point for each of the following: (i) severe (= 2) WMLs in the anterior or posterior periventricular white matter; (ii) ≥ 2 lacunes; and (iii) severe (= 2) central or cortical atrophy. Rather than quantifying single features of SVD, we selected this ordinal sum score since it quantifies the global burden of SVD from 0 (no imaging features of severe SVD) to 3 (all three severe imaging features of SVD). M.A.R was blinded to cognitive status before ICH and neuropathological findings.

For APOE genotype analysis, we obtained DNA from peripheral blood samples or cerebellar tissue stored in the LINCHPIN brain bank with the standard methods described.^29^ Investigators were masked to clinical, CT and pathological features during DNA extraction and genotyping.^29^ We classified the APOE genotype as APOE ε2 possession if participants had at least one ε2 allele or APOE ε4 possession if they had at least one ε4 allele.

### Neuropathological assessment

Two neuropathologists (C.S. and C.A.H.) assessed post-mortem brain tissue according to a standard operating procedure.^29^ The median interval between death and autopsy was 3 days [interquartile range (IQR) 2–4 days]. C.S. and C.A.H. rated samples from both hemispheres for non-CAA SVD,^34^ NFT,^35^ AP^36^ and CAA^37^ using validated rating scales. Macroscopic neuropathological assessment could not be masked to ICH location but microscopic assessment was masked to clinical and radiological findings including ICH location (unless the ICH was included on one of the pre-specified sample regions), cognitive status before ICH and CT SVD scores.

### Statistical analysis

We compared clinical and radiological characteristics between participants without cognitive impairment versus with cognitive impairment without dementia versus with dementia before ICH using the *χ*^2^ test (or Fisher’s exact test where appropriate) for categorical variables, and the Kruskal–Wallis test for non-normally distributed continuous variables. We used the Benjamini–Hochberg method to correct for multiple comparisons,^38^ with the false discovery rate set at 0.20.

We compared the severity of CAA, non-CAA SVD, NFT and AP in participants without cognitive impairment versus with cognitive impairment without dementia versus with dementia, stratified by ICH location (lobar versus non-lobar ICH). In a pre-specified sensitivity analysis, we excluded participants whose ICH location was classified as either ‘probable lobar’ or ‘probable deep’ according to the CHARTS rating tool.^30^ In two *post hoc* sensitivity analyses we firstly restricted the comparison of CAA, non-CAA SVD, NFT and AP by cognitive status to those participants who died within 180 days of their ICH and secondly compared the severity of NFT and AP by ICH location (irrespective of cognitive status).

We conducted all analyses in Stata version 11.2 or the statistical programming language R (version 4.0.3),^39^ with the use of finalfit package (version 1.0.3).^40^ Replication files are available on request.

## Results

Between June 2010 and August 2016, 125 adults (lobar ICH *n* = 71; non-lobar ICH *n* = 54) with first-ever spontaneous ICH underwent research autopsy after a first-ever ICH. One hundred and twenty-four participants had a diagnostic CT head before death and one patient was diagnosed with ICH at autopsy. The median interval between ICH onset and death was 10 days (IQR, 3–152 days) and the interval did not vary either by ICH location (lobar ICH median, 14 days; IQR, 3–270; and non-lobar ICH median, 9 days; IQR, 3–44 days; *P* = 0.268) or by cognitive status [without cognitive impairment median 9 days (IQR, 3–139 days); cognitive impairment without dementia median 8 days (1–110 days); dementia median 21 days (8–492); *P* = 0.111]. Twenty-two (18%) participants were able to provide demographic and clinical information themselves and in the remainder we obtained this information by interviewing their next-of-kin and from medical records.

The median age of the cohort was 82 years (IQR 76–86). 83 (66%) had no cognitive impairment; 21 (17%) had cognitive impairment without dementia and 21 (17%) had dementia before their ICH. Two participants (2%) with non-lobar ICHs were of black ethnicity and the remainder were white. 67 (54%) were female.

### Clinical characteristics of cohort, by ICH location

Of 71 participants with lobar ICH, 47 (66%) had no history of cognitive impairment, 12 (17%) had cognitive impairment without dementia and 12 (17%) had dementia before their ICH (Table 1).

**Table 1:** Baseline clinical features of participants with first-ever lobar ICH without prior cognitive impairment versus with cognitive impairment (but not dementia) versus with prior dementia (*n* = 71)

	No cognitive impairment (*n* = 47)	Cognitive impairment without dementia (*n* = 12)	Prior dementia (*n* = 12)	*P*
**Demographics**				
**Age, years**	81 (77–87)	83 (77–85)	83 (80–86)	0.861
**Sex (female)**	28 (60)	9 (75)	6 (50)	0.517
**Medical history**				
**History of depression**	4 (9)	1 (9)	4 (33)	0.087
**History of hypertension**	34 (72)	6 (50)	7 (58)	0.282
**History of ischaemic stroke**	5 (11)	3 (25)	2 (17)	0.426
**History of atrial fibrillation**	15 (32)	3 (25)	3 (25)	0.864
**History of diabetes**	5 (11)	2 (17)	1 (8)	0.855
**History of MI**	2 (4)	0 (0)	0 (0)	1
**Risk factors**				
**Smoking**				0.562
**Current**	9 (19)	0 (0)	2 (17)	
**Ex**	15 (32)	6 (50)	4 (33)	
**Never**	23 (49)	6 (50)	6 (50)	
**Alcohol intake per week**				0.616
**> 14 units**	6 (13)	1 (8)	0 (0)	
**< or = 14 units**	41 (87)	11 (92)	12 (100)	
**Apolipoprotein E (ApoE) genotype**				
**ApoE ε2 genotype^a^**	11 (23)	4 (33)	3 (25)	0.656
**ApoE ε4 genotype^a^**	13 (28)	4 (33)	6 (50)	0.302
**Drug history at the time of ICH**				
**Antiplatelet use**	21 (45)	8 (67)	5 (42)	0.354
**Anticoagulation use**	6 (13)	1 (8)	3 (25)	0.585
**Antihypertensive use**	28 (60)	4 (33)	4 (33)	0.115
**Statin use**	15 (32)	6 (50)	4 (33)	0.498
**Modified Rankin Score (mRS) before ICH**				**<** **0.001**
**0–2**	40 (85)	8 (67)	3 (25)	
**3–5**	7 (15)	4 (33)	9 (75)	

Data are *n* (%) or median (IQR). ^a^Data missing in 1 patient. Bold *P* values show statistically significant results adjusted using the Benjamini and Hochberg method. ICH, intracerebral haemorrhage; MI, myocardial infarction.

Of 54 participants with non-lobar ICH, 36 (66%) had no history of cognitive impairment, 9 (17%) had cognitive impairment without dementia and 9 (17%) had dementia before their ICH (Table 2).

**Table 2:** Baseline clinical features of participants with first-ever non-lobar ICH without prior cognitive impairment versus with cognitive impairment (but not dementia) versus with prior dementia (*n* = 54)

	No cognitive impairment (*n* = 36)	Cognitive impairment without dementia (*n* = 9)	Prior dementia (*n* = 9)	*P*
**Demographics**				
**Age, years**	81 (74–88)	76 (75–84)	86 (78–88)	0.216
**Sex (female)**	18 (50)	2 (22)	4 (44)	0.463
**Medical history**				
**History of depression**	8 (22)	2 (22)	2 (22)	1
**History of hypertension**	26 (72)	5 (56)	8 (89)	0.284
**History of ischaemic stroke**	4 (11)	0 (0)	3 (33)	0.100
**History of atrial fibrillation**	9 (25)	4 (44)	4 (44)	0.382
**History of diabetes**	4 (11)	2 (22)	2 (22)	0.614
**History of MI**	1 (3)	0 (0)	0 (0)	1
**Risk factors**				
**Smoking**				0.773
**Current**	7 (19)	1 (11)	2 (22))	
**Ex**	11 (31)	5 (56)	3 (33)	
**Never**	18 (50)	3 (33)	4 (44)	
**Alcohol intake per week**				0.257
**> 14 units**	9 (25)	1 (11)	0 (0)	
**< or = 14 units**	27 (75)	8 (89)	9 (100)	
**Apolipoprotein E (ApoE) genotype**				
**ApoE ɛ2 genotype^a^**	8 (22)	1 (11)	0 (0)	0.323
**ApoE ɛ4 genotype^a^**	11 (31)	2 (22)	4 (44)	0.686
**Drug history at the time of ICH**				
**Antiplatelet use**	15 (42)	2 (22)	4 (44)	0.700
**Anticoagulation use**	9 (25)	4 (44)	2 (22)	0.551
**Antihypertensive use**	20 (56)	4 (44)	4 (44)	0.778
**Statin use**	11 (31)	2 (22)	4 (44)	0.686
**Modified ranking score (mRS) before ICH**				**0.006**
**0–2**	25 (69)	6 (67)	1 (11)	
**3–5**	11 (31)	3 (33)	8 (89)	

Data are *n* (%) or median (IQR). ^a^Data missing in 1 patient who had cognitive impairment without dementia. This patient did not have a diagnostic CT (ICH diagnosis made at autopsy). Bold *P* values show statistically significant results adjusted using the Benjamini and Hochberg method. ICH, intracerebral haemorrhage; MI, myocardial infarction.

For both lobar and non-lobar ICH, the three groups of participants did not differ significantly in terms of their demographic variables, medical history, smoking or alcohol intake, antithrombotic medication, statin use or ApoE genotype at the time of ICH (Tables 1 and 2). Dependency before both lobar and non-lobar ICH as measured by the modified Rankin score was less common in adults without cognitive impairment and those with cognitive impairment without dementia compared to those with dementia (Tables 1 and 2).

13 (10%) participants had a recurrent ICH before death. About 10/13 (77%) of these participants had originally had a lobar ICH. 11/13 (85%) did not have a history of cognitive decline and the remaining 2 (15%) participants had cognitive impairment without dementia. Therefore, 11/83 (13%) participants without a history of cognitive decline and 2/21 (10%) participants with cognitive impairment (without dementia) had a recurrent ICH before death.

### Diagnosis and clinical phenotyping of dementia

Seventy-eight (62%) participants had an IQCODE. The proportion of participants with an IQCODE did not vary by cognitive status (without cognitive impairment – has IQCODE *n* = 51 [61%], cognitive impairment without dementia – has IQCODE *n* = 10 [48%], dementia –has IQCODE *n* = 17 [81%]; *χ*^2^ = 5.07, *P* = 0.09). Of 21, 19 (90%) with dementia and 16 of 21 (76%) with cognitive decline (without dementia) had a comprehensive care of the elderly or old age psychiatry assessment including an assessment of cognition before their ICH.

Of 12 participants in the lobar group with dementia before ICH, the most frequent syndrome was a probably mixed dementia (fulfilling criteria for both Alzheimer’s disease and vascular dementia; *n* = 5, 42%; Table 3). Two participants (17%) in the lobar ICH group fulfilled the criteria for Alzheimer’s disease. Of nine participants in the non-lobar group with dementia before ICH, the most frequent syndrome (*n* = 5, 56%) was probable vascular dementia. We were unable to make a syndrome diagnosis in 6 (29%) participants with dementia before ICH (Table 3). We describe selected clinical characteristics of those presenting with dementia before ICH including their IQCODE score, their pre-morbid dependency and whether they were taking cholinesterase inhibitors at the time of their ICH in the supplement (Supplementary Table 1). We did not have any participants meeting the criteria for dementia with Lewy bodies, Parkinson’s disease dementia or frontotemporal dementia.

**Table 3:** Clinical phenotyping of 21 participants with dementia before their ICH

	Lobar ICH (*n* = 12)	Non-lobar ICH (*n* = 9)
Alzheimer’s disease (NIA-AA criteria); *n* (%)	2 (17)	0 (0)
Probable vascular dementia (NINDS-AIREN criteria); *n* (%)	1 (8)	5 (56)
Probable mixed dementia, fulfilling criteria for Alzheimer’s disease and vascular dementia; *n* (%)	5 (42)	2 (22)
No syndrome diagnosis possible; *n* (%)	4 (33)	2 (22)

### Imaging characteristics

In the lobar ICH group, the three groups of participants did not differ in their ICH volumes or CT biomarkers of SVD (Table 4).

**Table 4:** Baseline diagnostic non-contrast brain CT features of participants with first-ever lobar ICH without prior cognitive impairment versus with cognitive impairment (but not dementia) versus with prior dementia (*n* = 71)

	No cognitive impairment (*n* = 47)	Cognitive impairment without dementia (*n* = 12)	Prior dementia (*n* = 12)	*P*
**ICH volume, mL**	18 (55–101)	50 (15–173)	51 (19–73)	0.785
**Multiple ICHs**	6 (13)	3 (25)	1 (8)	0.585
**Number of lacunes**	0 (0–0)	0 (0–0)	0 (0–0)	0.981
**≥ 2 lacunes**	4 (9)	0 (0)	2 (17)	0.384
**Anterior WMLs, *n* (%)**				
** 0**	11 (23)	0 (0)	1 (8)	0.101
** 1**	28 (60)	7 (59)	6 (50)	
** 2**	8 (17)	5 (41)	5 (42)	
**Posterior WMLs**				
** 0**	12 (25)	0 (0)	2 (17)	0.149
** 1**	5 (11)	4 (33)	2 (17)	
** 2**	30 (64)	8 (67)	8 (67)	
**Severe (= 2) anterior or posterior WMLs**	30 (64)	8 (67)	8 (67)	1.000
**Central atrophy**				
** 0**	14 (30)	4 (33)	2 (17)	0.473
** 1**	31 (66)	7 (59)	8 (67)	
** 2**	2 (4)	1 (8)	2 (17)	
**Cortical atrophy**				0.939
** 0**	11 (23)	3 (25)	2 (17)	
** 1**	27 (58)	6 (50)	7 (58)	
** 2**	9 (19)	3 (25)	3 (25)	
**CT SVD score**				0.266
** 0**	13 (28)	2 (17)	3 (25)	
** 1**	26 (55)	8 (67)	4 (33)	
** 2**	5 (11)	2 (17)	5 (42)	
** 3**	3 (6)	0 (0)	0 (0)	

Data are *n* (%) or median (IQR). ICH location and volume relate to the largest ICH if there were multiple acute ICHs on the diagnostic brain CT. CT, computed tomography; ICH, intracerebral haemorrhage; SVD, small vessel disease; WMLs, white matter lucencies.

In the non-lobar ICH group, severe cortical atrophy was less common in those without cognitive impairment (8/36, 22%) and cognitive impairment without dementia (0/9, 0%) versus dementia (5/9, 56%); *P* = 0.008; (Table 5). Although there were smaller proportions of adults with two or more lacunes in the groups without cognitive impairment and those with cognitive impairment without dementia before ICH in comparison to those with dementia, this did not reach statistical significance [no cognitive impairment *n* = 7 (19%), cognitive impairment without dementia *n* = 1 (12%), dementia *n* = 5 (56%), *P* = 0.061]. Severe anterior WMLs were also less common in participants without cognitive impairment before ICH (9/36, 25%) versus participants with cognitive impairment without dementia (3/8, 38%) versus dementia (6/9, 67%) but this did not reach statistical significance (*P* = 0.074).

**Table 5:** Baseline diagnostic non-contrast brain CT features of participants with first-ever non-lobar ICH without prior cognitive impairment versus with cognitive impairment (but not dementia) versus with prior dementia (*n* = 54)

	No cognitive impairment (*n* = 36)	Cognitive impairment without dementia (*n* = 9)	Prior dementia (*n* = 9)	*P*
**ICH volume, mL**	16 (8–37)	12 (6–26)	14 (11–16)	0.647
**Multiple ICHs**	3 (8)	0 (0)	0 (0)	1
**Number of lacunes^a^**	0 (0–1)	0 (0–1)	3 (0–3)	0.192
**≥ 2 lacunes^a^**	7 (19)	1 (13)	5 (56)	0.061
**Anterior WMLs, *n* (%)^a^**				
** 0**	3 (8)	2 (25)	0 (0)	0.074
** 1**	24 (67)	3 (38)	3 (33)	
** 2**	9 (25)	3 (38)	6 (67)	
**Posterior WMLs^a^**				
** 0**	11 (31)	2 (25)	2 (22)	0.149
** 1**	6 (17)	3 (38)	2 (22)	
** 2**	19 (52)	3 (38)	5 (56)	
**Severe (= 2) anterior or posterior WMLs^a^**	20 (56)	3 (38)	6 (67)	0.540
**Central atrophy^a^**				
** 0**	5 (14)	1 (13)	0 (0)	0.840
** 1**	21 (58)	4 (50)	5 (56)	
** 2**	10 (28)	3 (38)	4 (44)	
**Cortical atrophy^a^**				**0**.**008**
** 0**	2 (6)	0 (0)	2 (22)	
** 1**	26 (72)	8 (100)	2 (22)	
** 2**	8 (22)	0 (0)	5 (56)	
**CT SVD score^a^**				0.091
** 0**	10 (28)	3 (38)	1 (11)	
** 1**	13 (36)	3 (38)	0 (0)	
** 2**	10 (28)	2 (25)	6 (67)	
** 3**	3 (8)	0 (0)	2 (22)	

Data are *n* (%) or median (IQR). ^a^Data missing in 1 patient who had cognitive impairment without dementia. This patient did not have a diagnostic CT (ICH diagnosis made at autopsy). ICH location and volume relate to the largest ICH if there were multiple acute ICHs on the diagnostic brain CT. Bold *P* values show statistically significant results adjusted using the Benjamini and Hochberg method. CT, computed tomography; ICH, intracerebral haemorrhage; SVD, small vessel disease; WMLs, white matter lucencies.

### Neuropathological findings

Moderate or severe CAA was more common in adults with lobar ICH compared to those with non-lobar ICH (moderate or severe CAA: lobar: 43 [61%]; non-lobar 9 [17%] *χ*^2^ = 22.55 *P* ≤ 0.001). Moderate or severe non-CAA SVD was more common in adults with non-lobar ICH compared to adults with lobar ICH (moderate or severe non-CAA SVD lobar: 58 [82%]; non-lobar 53 [98%] *χ*^2^ = 6.78, *P* = 0.009).

The proportions of participants with moderate or severe CAA and non-CAA SVD in the three groups did not differ between those without cognitive impairment versus cognitive impairment without dementia versus dementia in either lobar ICH or non-lobar ICH (Tables 6 and 7).

**Table 6:** Neuropathological features of participants with first-ever lobar ICH without prior cognitive impairment versus with cognitive impairment (but not dementia) versus with prior dementia

	No cognitive impairment (*n* = 47)	Cognitive impairment without dementia (*n* = 12)	Prior dementia (*n* = 12)	*P*
**CAA**				0.231
** Absent/mild**	21 (45)	5 (42)	2 (17)	
** Moderate/severe**	26 (55)	7 (58)	10 (83)	
**Non-CAA SVD score**				0.741
** Mild**	10 (21)	1 (8)	2 (17)	
** Moderate/severe**	37 (79)	11 (92)	10 (83)	
**Braak staging^a^**	2 (2–3)	4 (2–6)	5.5 (4–6)	**0**.**004**
**Thal staging**	2 (1–2)	2 (2–3)	2.5 (2–3.5)	**0**.**033**

Data are *n* (%) or median (IQR). ^a^Data missing in 1 patient. Bold *P* values show statistically significant result. CAA, cerebral amyloid angiopathy; ICH, intracerebral haemorrhage; SVD, small vessel disease. Braak staging (32)—rating method for neurofibrillary tangle pathology. Thal staging (33)—rating method for amyloid plaque pathology.

**Table 7:** Neuropathological features of participants with first-ever non-lobar ICH without prior cognitive impairment versus with cognitive impairment (but not dementia) versus with prior dementia

	No cognitive impairment (*n* = 36)	Cognitive impairment without dementia (*n* = 9)	Prior dementia (*n* = 9)	*P*
**CAA**				0.221
** Absent/mild**	32 (89)	6 (67)	7 (78)	
** Moderate/severe**	4 (11)	3 (33)	2 (22)	
**Non-CAA SVD score**				1
** Mild**	1 (3)	0 (0)	0 (0)	
** Moderate/severe**	35 (97)	9 (100)	9 (100)	
**Braak staging**	2 (1–2)	2 (1–2)	5 (3–6)	**<** **0**.**001**
**Thal staging**	1 (0–1)	0 (0–2)	3 (2–3)	**0**.**002**

Data are *n* (%) or median (IQR). Bold *P* values show statistically significant result. CAA, cerebral amyloid angiopathy; ICH, intracerebral haemorrhage; SVD, small vessel disease. Braak staging (32)—rating method for neurofibrillary tangle pathology. Thal staging (33)—rating method for amyloid plaque pathology.

In adults with lobar ICH (Table 6), those without cognitive impairment before ICH had milder NFT pathology measured by median Braak stage (no cognitive impairment 2 [IQR 2–3] versus cognitive impairment without dementia 4 [2–6] versus dementia 5.5 [4–6]; *P* = 0.004) and milder AP pathology measured by median Thal stage (no cognitive impairment 2 [1–2] versus cognitive impairment without dementia 2 [2–3] versus dementia 2.5 [2–3.5]; *P* = 0.033).

In adults with non-lobar ICH (Table 7), those without cognitive impairment before ICH had milder NFT pathology measured by median Braak stage (no cognitive impairment 2 [1–2] versus cognitive impairment without dementia 2 [1–2] versus dementia 5 [3–6]; *P* < 0.001) and milder AP pathology measured by median Thal stage (no cognitive impairment 1 [0–1] versus cognitive impairment without dementia 0 [0–2] versus dementia 3 [2–3]; *P* = 0.002).

All results remained unchanged in the sensitivity analysis which excluded adults with ‘probable lobar’ (*n* = 6) and ‘probable non-lobar’ (*n* = 7) ICH.

When the analysis was restricted to those participants who died within 180 days of their ICH (*n* = 96; lobar ICH *n* = 52, non-lobar ICH *n* = 44), in the lobar ICH group there was no significant difference in the burden of AP between those without cognitive impairment (Thal stage median 2; IQR 1–2) versus those with cognitive decline without dementia (2 [2–2.5]) versus dementia (2 [2–3]); *P* = 0.100. Other results were unchanged (Supplementary Tables 2 and 3).

When the severity of NFT and AP were compared by lobar versus non-lobar ICH (irrespective of cognitive status), those with lobar ICH had both more severe NFT pathology as measured by median Braak stage (3 [IQR 2–5]) in comparison to those with non-lobar ICH (2 [1–3]); *P* = 0.006, and more severe AP pathology (lobar ICH median Thal stage 2 [1–3]; non-lobar ICH 1 [0–2]; *P* = 0.002).

## Discussion

In this prospective community-based study, we found that one-third of the cohort had cognitive impairment or dementia before their ICH. Adults with dementia before ICH were more dependent but other clinical characteristics were similar between groups. A larger proportion of adults with dementia before a non-lobar ICH had severe cortical atrophy. The cognitive status of adults with moderate or severe CAA or non-CAA SVD did not vary before lobar versus non-lobar ICH. Adults with lobar ICH had more severe NFT and AP but irrespective of ICH location, adults who did not have cognitive impairment before their ICH had milder NFT and AP pathology.

This study’s findings are consistent with others. The prevalence of cognitive impairment or dementia in our cohort is similar to a recent systematic review which found that cognitive impairment and dementia affected about one-third of participants before ICH.^2^ In keeping with other studies,^18,41^ we found that people with dementia had a higher level of dependency before their ICH compared with people with cognitive impairment (but without dementia) and people without dementia. Unlike two other studies which found that prior stroke or transient ischaemic attack^18,42^ and ischaemic heart disease^18^ were associated with dementia before ICH, we did not find any difference in clinical characteristics between people with cognitive impairment or dementia and people without, possibly because of a lack of power. In keeping with one study,^18^ we found that people with dementia before their non-lobar ICH had more cortical atrophy which may be a direct effect of neurodegeneration or reflect a reduction in white matter volume caused by moderate–severe non-CAA SVD,^43^ which was more common in the non-lobar ICH group. Two studies^41,44^ have reported that larger ICH volumes are associated with dementia before ICH. We found no such association but this may be because people in our cohort who donated brain tissue tended to have larger ICH volumes.^23^

Our study adds to what is known about the pathology underlying dementia before ICH. We found that irrespective of ICH location, adults without cognitive impairment before ICH had milder NFT and AP pathology. Although moderate or severe CAA was more common in lobar ICH and moderate or severe non-CAA SVD was more common in non-lobar ICH, reflecting the known associations of CAA with lobar ICH^9^ and non-CAA SVD with non-lobar ICH, the severity of CAA and non-CAA SVD did not differ according to dementia status in adults with lobar or non-lobar ICH in our cohort. This may be because our sample size was too small or because CAA and non-CAA SVD are intermediate processes, either or both of which contribute to the formation of white matter lesions, Alzheimer disease neuropathologic change, and the clinical manifestations of dementia rather than directly causing dementia themselves.^45^ Alternatively, given that this is an autopsy cohort, participants were likely to have larger ICH volumes^23^ and those without cognitive impairment may have had more severe CAA and non-CAA SVD but possessed an unknown characteristic protective against dementia.

There is general agreement that dementia is associated with mixed vascular and neurodegenerative pathologies^7,46-48^ and that vasculopathies such as CAA and non-CAA SVD aggravate neurodegenerative pathologies.^3,7,49^ In our cohort, those with lobar ICH had more severe Alzheimer's disease neuropathologic change reflecting the known associations between CAA, lobar ICH and Alzheimer's disease.^18,21^ However, our observation that people without cognitive impairment had milder NFT and AP irrespective of ICH location, suggests that NFT and AP may increase susceptibility to pre-ICH cognitive impairment and dementia. AP burden did not differ by cognitive status in the lobar ICH group when the analysis was restricted to people who died within 180 days of ICH onset but we may have lacked the power to detect a difference between groups, especially since people without cognitive impairment in the lobar ICH group had a higher median AP burden compared to people without cognitive impairment in the non-lobar ICH group. Although ICH itself may increase levels of amyloid-beta,^50^ the interval between ICH onset and death did not differ by cognitive status and we classified cognitive status at presentation blinded to neuropathology, making it less likely that the neuropathological findings are due to measurement bias.

This study has many merits. This is the first-ever prospective community-based study of the brain pathologies underlying dementia before ICH which integrated both imaging and neuropathology findings. We sought to enrol consecutive people who we were able to approach for consent and levels of missing data are low, reducing selection bias and improving the generalizability of the findings. Participants with recurrent ICHs at the time of inception into the study were not eligible (since we wished to classify dementia at a uniform time point thereby ensuring that dementia preceded the ICH) and most participants who went on have recurrent ICH during the course of the study did not have cognitive decline before their first ICH reducing the likelihood that the differences in neuropathologic findings between the groups arose because of a greater burden of SVD in the group who subsequently developed recurrent ICH. We minimized information bias by using standardized assessments of both CT SVD biomarkers and neuropathologic findings with a neuroradiologist blinded to neuropathologic findings and the neuropathologist blinded to clinical details and CT SVD findings. We minimized the misclassification bias of dementia by using well-established diagnostic criteria and a short form of the IQCODE which is a valid measure of pre-existing dementia in stroke patients.^51,52^

This study has some weaknesses. Given that this is an autopsy cohort, adults were likely to have more severe SVD^29^ and neuropathologic change compared with ICH survivors. Nevertheless, our finding of greater cortical atrophy in people with dementia before non-lobar ICH is consistent with previous studies. We had insufficient information on education level or socioeconomic status, both of which are risk factors for the development of dementia. We cannot exclude the possibility that some participants who had not undergone a formal cognitive assessment before their ICH had mild cognitive impairment but were misclassified as having no cognitive impairment. However, the prevalence of cognitive impairment and dementia in our cohort is similar to previous studies^2^ making it less likely that the findings are subject to misclassification bias. Our cohort was almost entirely white, so we were unable to explore variation between ethnic groups. In this study, we did not assess the contribution of other neuropathologic changes such as Lewy bodies, non-NFT tauopathies, or phosphorylated TAR-DNA binding protein 43 although our clinical phenotyping of people with dementia did not reveal any participants with a clinical diagnosis of Lewy-body dementia of frontotemporal dementia. These participants did not undergo ‘antemortem’ MRI which may have provided detailed information regarding manifestations of SVD such as microbleeds. However, this study reflects real-world practice in that CT is the most commonly used imaging modality to diagnose ICH, and frequently, those with ICH are too unwell to undergo MRI.

## Conclusion

In summary, we have shown that dementia before the first-ever non-lobar ICH is associated with more severe cortical atrophy and that irrespective of ICH location adults who did not have cognitive impairment before their ICH had milder NFT and AP. Future large prospective studies in different ethnic populations should explore if these findings can be reproduced and assess the risk factors which contribute to dementia before and after ICH.

## Supplementary Material

fcae275_Supplementary_Data

## Data Availability

A fully anonymized version of the dataset used for analysis with a data dictionary will be available on Edinburgh Datashare (https://datashare.ed.ac.uk/) for other researchers to apply to use 1 year after publication. Further enquiries can be directed to the corresponding author.
